# Characteristic of Endometrial Stromal Sarcoma by Algorithm of Potential Biomarkers for Uterine Mesenchymal Tumor

**DOI:** 10.3390/cimb45080390

**Published:** 2023-07-25

**Authors:** Takuma Hayashi, Kenji Sano, Nobuo Yaegashi, Kaoru Abiko, Ikuo Konishi

**Affiliations:** 1 Cancer Medicine, National Hospital Organization Kyoto Medical Centre, Kyoto 612-8555, Japan; 2 Department of Medical R&D Promotion Project, Japan Agency for Medical Research and Development (AMED), Tokyo 103-0022, Japan; 3 Department of Pathology, Shinshu University Hospital, Nagano 390-8621, Japan; 4 Section of Assistant Director, Sendai Red Cross Hospital, Miyagi 982-8501, Japan; 5 Department of Obstetrics and Gynecology, National Hospital Organization Kyoto Medical Centre, Kyoto 612-8555, Japan; 6 Department of Obstetrics and Gynecology, Graduate School of Medicine, Kyoto University, Kyoto 606-8507, Japan

**Keywords:** endometrial stromal sarcoma, mesenchymal tumor, leiomyosarcoma, leiomyoma

## Abstract

The benign tumor uterine leiomyoma (UL) develops from the smooth muscle tissue that constitutes the uterus, whereas malignant tumor uterine sarcoma develops from either the smooth muscle tissue or stroma and is different from UL and endometrial cancer. Uterine sarcoma is broadly classified into three types: uterine leiomyosarcoma, endometrial stromal sarcoma (ESS), and carcinosarcoma. Although uterine leiomyosarcoma and ESS are both classified as uterine sarcoma, they significantly differ in terms of their sites of occurrence, symptoms, and treatment methods. Uterine leiomyosarcoma develops from the muscle tissue constituting the wall of the uterus and accounts for approximately 70% of all uterine sarcoma cases. In contrast, ESS develops from the stromal tissue beneath the endometrium and accounts for approximately 25% of all uterine sarcoma cases. ESS is classified as either low grade (LG) or high grade (HG). This case report aimed to highlight the importance of histopathologic examinations based on surgical specimens. Herein, we reported the case of a 45-year-old woman suspected of having submucosal leiomyoma of the uterus based on imaging results. Transvaginal ultrasonography and endometrial biopsy or partial dilation and curettage were performed. Contrast-enhanced magnetic resonance imaging (MRI) revealed a 32-mm mass projecting from the posterior wall of the uterus into the uterine cavity. T2-weighted imaging revealed a low signal within the mass; thus, submucosal UL was suspected. Histopathologic examination of surgical specimens obtained from a patient suspected of having submucosal UL after contrast-enhanced MRI indicated that the patient had ESS. Despite the remarkable advancements in medical imaging technology, the accuracy of contrast-enhanced MRI for detecting uterine mesenchymal tumors is limited. Therefore, histopathologic diagnosis based on surgical specimens should be performed when medical grounds for diagnosing a benign tumor on contrast-enhanced MRI are lacking.

## 1. Introduction

The benign tumor uterine leiomyoma (UL) derives from the smooth muscle tissue that constitutes the uterus [1,2]. UL is most frequently seen in relatively young-to-postmenopausal women [1] and is often found incidentally during medical examinations in the absence of any particular symptoms. Depending on the site of origin, UL is classified as either a subserosal, intramural, or submucosal leiomyoma [1,3] and can exhibit hemorrhage, necrosis, calcification, and edema. Symptoms of UL include hypermenorrhea, menstrual pain, palpable abdominal mass, and anemia, among others. When a UL enlarges, it puts pressure on the surrounding organs, and symptoms such as frequent urination, dysuria, constipation, and lumbago are observed. Infertility and miscarriage can also result [4]. A patient’s chief complaint will depend on the site of onset and the size and number of ULs. For example, compared with other leiomyomas, a submucosal leiomyoma tends be associated with excessive menorrhagia, prolonged menstruation, and menstrual pain even when the tumor is small, and the patient tends to be prone to anemia [5]. Surgery is the typical treatment for patients with symptoms; however, when asymptomatic, patients with UL are often simply watchfully followed.

UL is diagnosed by pelvic exam, ultrasonography, and if necessary, contrast-enhanced magnetic resonance imaging (MRI) [6]. If the findings obtained from ultrasonography or contrast-enhanced MRI are not typical for UL or if the patient is experiencing rapid or postmenopausal mass enlargement, the rare malignant tumor uterine sarcoma, which also derives from uterine smooth muscle, should be added to the differential [7]. Uterine sarcoma is broadly classified into two types—uterine leiomyosarcoma and endometrial stromal sarcoma (ESS) [8]—which have different treatment approaches. According to the WHO classification of tumors of the uterine corpus 2020, carcinosarcomas are classified as endometrial epithelial tumors and precursors. [3].

The uterine tissues from which ESS and uterine leiomyosarcoma develop are different [9]. Uterine leiomyosarcoma arises in the smooth muscle tissue, and ESS arises in the endometrial or stromal tissue. Moreover, these two tumor types are associated with different ages of onset. Uterine leiomyosarcoma is typically found in women 50–55 years of age. ESS is typically found in premenopausal women in their 40s and can classified as either low-grade endometrial stromal sarcoma (LG-ESS) or high-grade endometrial stromal sarcoma (HG-ESS). Many cases of LG-ESS respond well to hormone therapy and have a favorable survival prognosis [10]. For the far rarer HG-ESS tumors, hormone therapy is not recommended [10]; patients, therefore, often receive chemotherapy, but the efficacy of that approach has not been confirmed. In uterine leiomyosarcoma, prognosis is typically poor, with the risk of recurrence and metastasis being significantly high. The first-line treatment for uterine leiomyosarcoma is surgical excision. Unfortunately, no low-toxicity and high-efficacy antitumor agents and radiation therapy for uterine leiomyosarcoma have so far been found [11].

A 45-year-old woman suspected of having a submucosal leiomyoma of the uterus based on the results of imaging performed at a nearby hospital was referred to our clinical department. Contrast-enhanced MRI had revealed a 32 mm mass projecting from the posterior wall of the uterus into the uterine cavity. MRI T2-weighted imaging (T2WI) revealed a low signal within the mass, and so a submucosal UL was suspected. However, contrast-enhanced diffusion-weighted MRI performed by our medical staff revealed a high signal in the area of the mass, suggesting the possibility of malignancy. Subsequent histopathology of the surgically resected specimen resulted in a diagnosis of ESS. Although medical imaging technology has progressed remarkably, the accuracy of contrast-enhanced MRI for detecting uterine mesenchymal tumors is limited. Histopathologic diagnosis based on surgical specimens should therefore be performed when medical grounds for diagnosing a benign tumor in contrast-enhanced MRI are lacking. This case report aimed to highlight the importance of histopathologic examinations based on surgical specimens. Herein, we reported the case of a 45-year-old woman suspected of having submucosal leiomyoma of the uterus based on imaging results.

## 2. Materials and Methods

### 2.1. Immunohistochemistry

Staining for caveolin-1, cyclin B, cyclin E1, large multifunctional protease 2 (LMP2)/β1i, Ki-67, desmin, and myogenin was performed on serial tumor sections obtained from patients with uterine mesenchymal tumors (Supplementary Material S1). The monoclonal antibody for cyclin E1 (CCNE1/2460) was purchased from Abcam (Cambridge Biomedical Campus, Cambridge, UK), and the monoclonal antibody for Ki-67 (clone MIB-1) was purchased from Dako Denmark A/S (DK-2600 Glostrup, Denmark). The monoclonal antibodies for desmin (clone RM234) and for myogenin (clone MGN185) were purchased from GeneTex, Inc. (Irvine, CA, USA). The monoclonal antibodies for caveolin-1, cyclin B1, and LMP2/β1i were purchased from Santa Cruz Biotechnology Inc. (Santa Cruz, CA, USA). All immunohistochemistry used the avidin–biotin complex method as previously described [12,13]. Briefly, one representative 5 mm tissue section was cut from the paraffin-embedded radical hysterectomy specimen obtained from each patient with a uterine mesenchymal tumor. The sections were incubated first with a biotinylated secondary antibody (Dako, DK-2600 Glostrup, Denmark) and then with streptavidin complex (Dako). The completed reaction was developed using 3,39′-diaminobenzidine tetrahydrochloride hydrate (DAB), and the slide was counterstained with hematoxylin. Normal myometrium portions in the specimens were used as positive controls. Tissue sections incubated with normal rabbit immunoglobulin G instead of the primary antibody were used as negative controls. Brown DAB staining revealed the expression of cyclin E and Ki-67. Normal rabbit or mouse antiserum was the negative control for the primary antibody. The DAB-stained tissue was scanned in its entirety using a digital microscope (BZ-X800: Keyence Corporation, Osaka, Japan). Black dots indicate the expression of cyclin E and Ki-67. Normal rabbit or mouse antiserum was used as the negative control for the primary antibody.

### 2.2. Pathogenic Variants of Patient’s Tumor and Human Uterine Leiomyosarcomas

Pathogenic variants of patient’s tumor and human uterine leiomyosarcomas are identified by the FoundationOne^®^ CDx tissue examination (Foundation Medicine, Inc., Cambridge, MA, USA) with patient tissues resected by surgical treatment.

### 2.3. Ethics Approval and Consent to Participate

Shinshu University approved the experiments (approval no. M192). All experiments using human tissue were conducted at the National Hospital Organization, Kyoto Medical Center (approval no. NHO H31-02) in accordance with institutional guidelines issued on 17 August 2019 by the Central Ethics Review Board of the National Hospital Organization Headquarters (Tokyo, Japan) and Shinshu University (Nagano, Japan). The authors attended educational lectures on medical ethics in 2020 and 2021, supervised by the Japanese government (completion numbers AP0000151756, AP0000151757, AP0000151769, and AP000351128). Consent to participate in this clinical research was required. After being briefed on the clinical study and agreeing with the clinical research objectives, participants signed consent forms. The authors attended a seminar on the ethics of experimental research using small animals on 2 July 2020, and 20 July 2021. The code number of the ethical approval for experiments with small animals was KMC R02-0702.

## 3. Case Description

On 14 March 2021, a 45-year-old woman with suspected submucosal UL was referred to our general clinical facility. At that time, contrast-enhanced MRI had revealed a 1.9 cm submucosal mass suspected to be the UL. Blood tests revealed a serum hemoglobin concentration of 11.6 g/dL and carbohydrate antigen 19-9 (CA19-9) (Carbohydrate antigen 19-9 (CA19-9) is associated with cancers of the colon, stomach, and bile duct. Elevated serum CA19-9 can indicate advanced cancer of the pancreas or an ovarian sclerosing stromal tumor, but is also associated with noncancerous conditions including gallstones, pancreatitis, cirrhosis of the liver, and cholecystitis [14,15].) and 72-4 values of 8 and 1.5 respectively. On the patient’s return to our general clinical facility on 10 October 2021, for further testing, contrast-enhanced MRI revealed a 3.9 cm mass under the uterine mucosa, suggesting that the tumor had enlarged over the elapsed 6 months. Based on contrast-enhanced T1-weighted imaging, the possibility of malignancy could not be ruled out. Thus, based on the contrast-enhanced MRI results and blood tests, we believed that the mass might be a uterine mesenchymal tumor rather than a UL. In January 2022, the patient underwent a laparoscopic total hysterectomy and bilateral salpingo-oophorectomy.

### 3.1. Details of the Contrast-Enhanced MRI

The contrast-enhanced MRI had revealed a mass of approximately 32 mm protruding from the posterior wall of the uterus into the lumen. On contrast-enhanced T2WI (sagittal high-pass; SAG H=F), the mass was observed to be of moderate-to-low intensity, suggesting a submucosal UL (Supplementary Figure S1A). In contrast, on diffusion-weighted imaging (echo-planar two-dimensional sequence; Ep2d), the mass was observed to have a high signal (Supplementary Figure S1B).

The T2WI also revealed bilateral ovarian shading. On T2WI (transverse relaxation time), bilateral ovarian cystic lesions with a fat-suppressed high signal (30 mm in the right ovary, and 35 mm in the left ovary) were observed (Supplementary Figure S1C). These masses were presumed to be endometriotic cysts. No significant lymphadenopathy was detected; however, a small ascites accumulation was noted.

### 3.2. Details of the Histopathologic Examination

Supplementary Figure S2A,B presents the macroscopic findings of the excised specimen. A solid white nodule measuring 4.0 cm can be observed within the wall of the uterine corpus.

On histopathology, the uterine mass was observed to be composed of short, spindle-shaped cells with a high nuclear/cytoplasmic ratio, surrounded everywhere by small spiral artery-like blood vessels. The cells constituting the mass demonstrated a mild degree of nuclear atypia. The number of mitotic figures was approximately one per high-power field, and no cell necrosis was observed. At the margin of the mass, tumor cells demonstrated tongue-like extensions into the surrounding normal smooth muscle tissue and blood vessels. Based on those histopathologic findings, the tumor was diagnosed as an LG-ESS.

**Figure 1:**
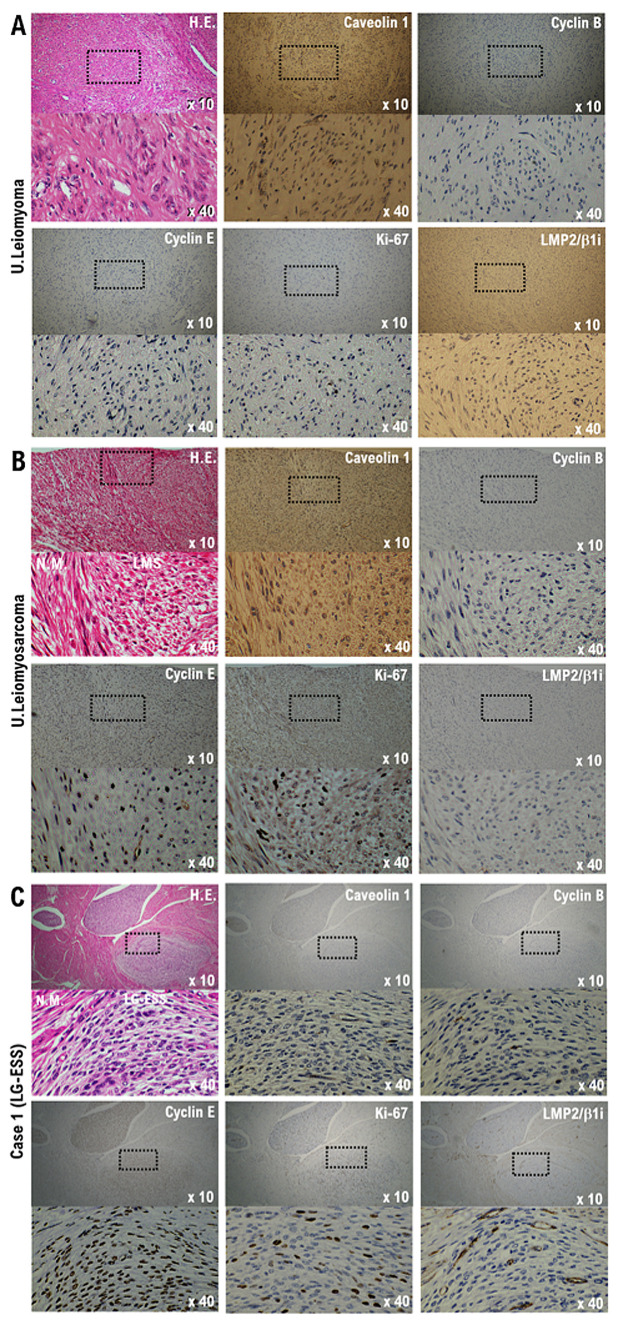
Differential expression of potential biomarkers cyclin B, cyclin E, caveolin-1, Ki-67, and LMP2/β1i in samples of normal myometrium, uterine (U.) leiomyoma, U. leiomyosarcoma, and our patient’s uterine tumor (Case 1). Immunohistochemistry for all specimen sections was performed using appropriate monoclonal antibodies and standard procedures. (**A**) The low-power (×10) view of leiomyoma shows a well-circumscribed tumor nodule in the myometrium; broad spindle cell fascicles are evident. In the high-power (×40) view, the spindle cells have bland cytologic features, with elongated nuclei and fine nuclear chromatin. (**B**) The low-power (×10) view of uterine epithelioid leiomyosarcoma shows a mass having an irregular interface with the myometrium; the constituent cells are round to polygonal, with granular eosinophilic cytoplasm. Significant nuclear atypia and mitoses are easily found. In the high-power view (×40), the tumor cells are round to ovoid and have eosinophilic granular cytoplasm and irregularly shaped nuclei. (**C**) In the low-power (×10) view, normal uterine smooth muscle differentiation is seen as a starburst morphology, with collagen bands radiating toward the periphery of the low-grade endometrial stromal sarcoma nodule, with its embedding round cells in a background of endometrial stromal neoplasia. The tumor is invading lymphatic vessels. In the high-power (×40) view, tumor cells can be observed to have a morphology quite different from that of normal uterine smooth muscle cells. The bottom photo is an enlargement of the dashed box in the top photo. Low-grade endometrial stromal sarcoma differentiation is evident. H.E., hematoxylin and eosin.

**Figure 2:**
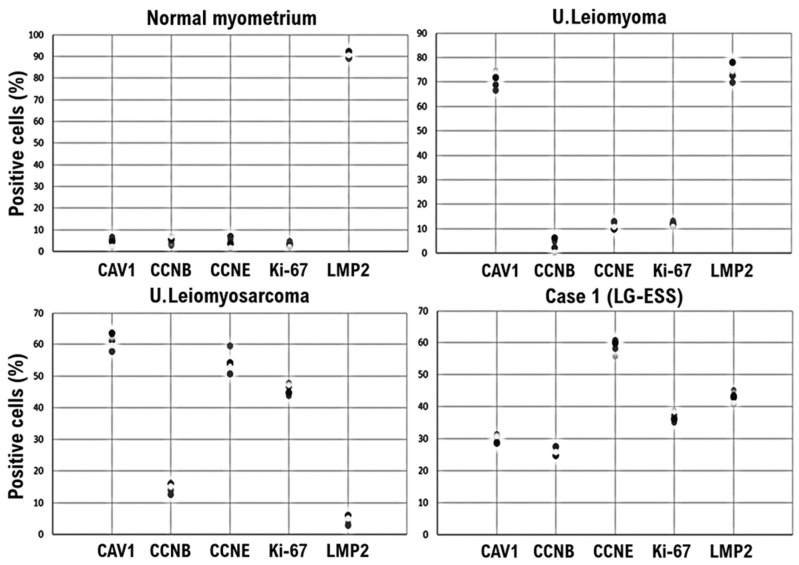
LMP2/β1i-positive endometrial stromal tumor cells in our patient’s uterine tumor (Case 1), contrasted with normal myometrium and uterine (U.) leiomyoma. Immunohistochemistry for all five randomly selected specimen sections was performed using appropriate monoclonal antibodies and standard procedures. In a 40× view, the positivity rates of the five factors were calculated for the four specimens and are presented in a scatterplot. CAV1, caveolin-1; CCNB, cyclin B; CCNE, cyclin E; LMP2, LMP2/β1i; LG-ESS, low-grade endometrial stromal sarcoma.

A molecular pathology analysis of multiple surgical specimens that used index markers for various soft tissue tumors, including candidate biomarkers for uterine leiomyosarcoma, clearly confirmed that caveolin, a candidate biomarker for uterine mesenchymal tumors, was expressed in both UL and uterine leiomyosarcoma (Figure 1A,B and Figure 2) [16]. Mild expression of caveolin was observed in our patient’s tumor (Figure 1C and Figure 2). Mild expression of cyclin B, considered a potential biomarker for malignant tumors, and strong expression of cyclin E and Ki-67, candidate biomarkers for malignant mesenchymal tumors, were confirmed in multiple specimens of uterine leiomyosarcoma and uterine tumors (Figure 1B,C and Figure 2). In published research reports, spontaneous onset of uterine leiomyosarcoma has been observed in mice deficient in LMP2/β1i, a subunit of the immunoproteasome [17,18]. In human uterine leiomyosarcoma, LMP2/β1i expression is significantly reduced (Figure 1B and Figure 2) [19]; however, as in normal uterine smooth muscle and UL, strong expression of LMP2/β1i was observed in our patient’s uterine tumor (Figure 1A,C and Figure 2). The foregoing findings suggested that our patient’s tumor was malignant, but that the possibility of uterine leiomyosarcoma was low.

**Figure 3:**
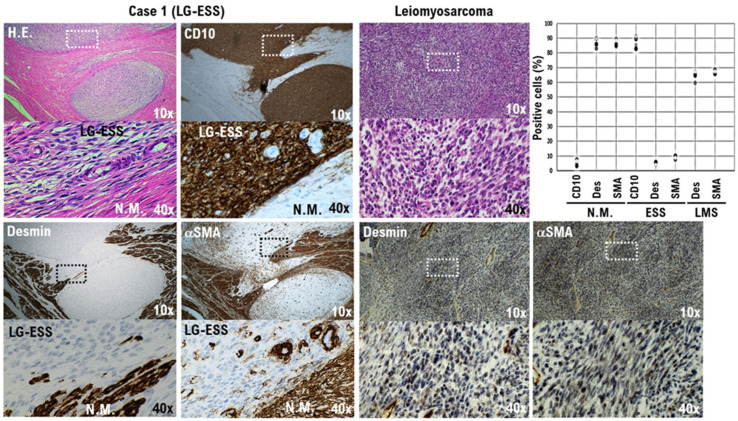
CD10-positive low-grade endometrial stromal sarcoma (LG-ESS) cells in our patient’s tumor (Case 1). The differential expression of the potential biomarkers desmin and α-SMA in the sarcoma cells, normal myometrium, and uterine leiomyosarcoma is presented. Immunohistochemistry for all specimen sections was performed using appropriate monoclonal antibodies and standard procedures (right lower panel). The low-power (10×) view at the farthest right in the first row shows the irregular interface of a uterine epithelioid leiomyosarcoma with normal myometrium. The tumor is observed to consist of round-to-polygonal cells having granular eosinophilic cytoplasm, with significant nuclear atypia and mitoses being easily found. In the accompanying high-power (40×) view, the tumor cells are round to ovoid, with eosinophilic granular cytoplasm and irregularly shaped nuclei. The low-power views on the left in the first row show the low-grade endometrial stromal sarcoma cells of our patient’s tumor. The cells are an admixture of round, polygonal, bizarre, and spindle types, with marked atypia and the occasional presence of giant cells. A tongue-like pattern of infiltration consisting of irregular islands of purple cells lacking an associated stromal response is evident. In the accompanying high-power views, the tumor cells can be seen to have a morphology quite different from that of normal uterine smooth muscle cells. In a 40× view, the positivity rates of desmin and α-SMA were calculated for the three specimens and are presented in a scatterplot. The bottom photo is an enlargement of the dashed box in the top photo.

Desmin, myoglobin, myogenin, MyoD1, α-smooth muscle actin (α-SMA), and hyperinsulinemic hypoglycema familial-35 (HHF-35), among others, are used as markers for myogenic tissue [20]. Cluster of differentiation 10 (CD10) is expressed in cells that constitute the endometrial stromal tissue, which are difficult to identify as epithelial cells [21]. Desmin and α-SMA are expressed in the myogenic cells of uterine smooth muscle. Immunohistochemical staining of our patient’s surgical specimen revealed a strong expression of CD10 in the patient’s tumor. However, CD10 expression was not detected in normal uterine smooth muscle tissue (Figure 3 and Figure S2C). Desmin was not observed to be strongly expressed in the patient’s uterine tumor (Figure 3 and Figure S2C). However, strong expression of desmin was observed in normal uterine smooth muscle tissue and in uterine leiomyosarcoma tissue (Figure 3 and Figure S2C). Similarly, α-SMA was not observed to be strongly expressed in the patient’s uterine tumor (Figure 3 and Figure S2C), but strong expression of α-SMA was observed in normal uterine smooth muscle tissue and uterine leiomyosarcoma tissue (Figure 3 and Figure S2C). Based on those observations, the patient’s tumor was considered to originate from endometrial stromal cells rather than from uterine smooth muscle cells. 

In clinical studies conducted by our research group to identify candidate factors of biomarker in various uterine mesenchymal tumor tissues, the expression status of each candidate factor in the tumor tissue of patients thought to have endometrial stromal sarcoma was investigated. It was revealed that the expression pattern of each candidate factor in the tissue of the patient’s tumor considered to be endometrial stromal sarcoma was different from the expression pattern of each candidate factor in the tissue of uterine leiomyosarcoma (Table 1).

### 3.3. Details of the Cancer Genome Examination

Pathogenic variants were examined by cancer genomic testing (FoundationOne^®^ CDx tissue, Foundation Medicine, Inc., Cambridge, MA, USA) using patient tissue resected by surgical treatment. As a result of examination of FoundationOne^®^ CDx tissue, microsatellite status; stable, tumor mutation burden (TMB); 5 Muts/Mb, *erb-b2 receptor tyrosine kinase 2 (ERBB2)*; and copy number 41, *trancher collins syndrome 2* (*TCS2)* Q1148* (allele frequency 43.4%), *tumor protein 53 (TP53)*, and t211I (allele frequency 54.9%) were detected as pathogenic variants (Supplementary Table S1). On the other hand, in our clinical research, from the analysis results of FoundationOne^®^ CDx tissue using uterine leiomyosarcoma tissue, many uterine leiomyosarcoma cases have been shown to harbor pathogenic variants in ATRX chromatin remodeler (ATRX), TP53, cyclin E1 (CCNE1), mediator complex subunit 12 (MED12), mouse double minute 2 homolog (MDM2), Large Multifunctional Protease 2 (LMP2)/β1i, etc (Supplementary Table S1). The genetical results obtained by the examination of FoundationOne^®^ CDx tissue showed that pathogenic variants of the tumor tissue obtained from the patient was distinct from those of uterine leiomyosarcoma. As for 2022, the number of patients with uterine leiomyoma or uterine sarcoma in our medical institution is shown in the Supplemental Table S2.

## 4. Discussion and Conclusions

Most uterine sarcomas (40–50%) are diagnosed as leiomyosarcoma, followed by LG-ESS, HG-ESS, undifferentiated uterine sarcoma, and adenosarcoma of the uterus [22]. Using contrast-enhanced MRI or other clinical examinations to determine the degree of malignancy or diagnose a mass found in uterine smooth muscle tissue is not easy [23]. Contrast-enhanced MRI in our patient revealed a 32 mm mass projecting from the posterior wall of the uterus into the uterine cavity. Based on the low signal observed within the mass on sagittal high-pass T2WI, a submucosal UL was suspected (Supplementary Figure S2A). However, diffusion-weighted imaging revealed a high signal within the mass, suggesting malignancy (Supplementary Figure S2B). After a total laparoscopic hysterectomy and bilateral salpingo-oophorectomy, histopathologic examination of the resected specimen demonstrated strong expression of CD10, a molecular marker for endometrial stromal cells. Moreover, desmin and α-SMA, known molecular markers of uterine smooth muscle cells, were not observed to be strongly expressed. Based on the MRI results and histopathology, the tumor was diagnosed as deriving from endometrial stromal cells.

The annual incidence of uterine sarcoma is 1–2 per 100,000 women. Uterine sarcoma is relatively rare, accounting for approximately 0.5–2.0% of all cases of uterine mesenchymal tumors [24]. Further, 50% of all uterine sarcoma cases recur within 2 years, even in patients with early-stage cancer (stage I). Furthermore, uterine sarcoma often exhibits distant metastasis, indicating its extremely poor prognosis. Approximately 90% of all patients with stage II or higher uterine sarcoma experience recurrence within 2 years [24]. Therefore, an accurate differential diagnosis of uterine leiomyoma and uterine sarcoma can greatly influence the treatment strategy. However, conventional diagnostic imaging methods (ultrasound tomography, CT, and MRI) do not provide sufficient accuracy for diagnosing uterine mesenchymal tumors. Among these methods, pelvic contrast-enhanced MRI has a relatively high diagnostic rate. When rapid tumor growth, hemorrhagic necrosis on MRI T1-weighted imaging, diffuse lobulated high-intensity mass on MRI T2-weighted imaging, tendency to infiltrate around the tumor, and vascular proliferation are observed, uterine sarcoma should be strongly suspected. However, MRI alone does not provide satisfactory results as a preoperative diagnostic method.

If symptoms suggesting uterine leiomyosarcoma or ESS are observed, transvaginal ultrasonography and partial cervical dilatation and curettage or histopathology of an endometrial biopsy specimen is usually performed. However, limitations in the detection sensitivity of those clinical examinations make an accurate diagnosis difficult [25]. In the clinic, ESS or uterine leiomyosarcoma might, therefore, more often be incidentally detected by histopathologic examination of specimens obtained from hysterectomy or enucleation.

ESS has a cytologic appearance similar to that of normal endometrial stromal cells. As part of our differential, histopathology revealed that the tumor cells had a high nuclear/cytoplasmic ratio, irregular nuclei with notches and constrictions, increased amounts of chromatin, and many mitotic figures [26,27]. The characteristic histopathology of ESS includes naked nucleate and stoma cells with small nucleoli [26,27]. However, it has been established that the lower the degree of malignancy, the weaker the cell atypia, and the fewer the number of cells with mitotic figures. Making a diagnosis based solely on findings from individual cells is therefore difficult. In general practice, when cell necrosis is not observed during the cytologic examination of the uterine body, and poorly atypical cells and clumps are observed, either atypical endometrial hyperplasia or benign endometrial hyperplasia is suspected. However, in HG-ESS, routine somatic cytology might reveal tumor cells; thus, cytology should be carefully performed. Although ESS is currently a rare tumor, the suspicion is that cases might increase in the future as the Japanese population ages. Careful assessment of non-epithelial cells is therefore necessary in endometrial cytology. Based on the contrast-enhanced MRI in our patient, the mass observed under the uterine mucosa was thought to possibly be a submucosal UL. However, histopathology of the surgical specimen revealed an ESS. The characteristic shadows of ESS are difficult to observe on contrast-enhanced MRI.

MRI, especially contrast-enhanced MRI, is indispensable for distinguishing between benign and malignant tumors in the uterine corpus and for determining the tumor’s tissue type. Compared with ultrasonography, MRI can more clearly visualize the characteristics of malignancy. T2WI, including a dynamic contrast-enhanced approach, is suitable for detecting small tumors. On T2WI, endometrial tumors are often less intense than normal endometrium and slightly more intense than myometrium. However, unlike UL, ESS does not appear as a localized mass with clear margins, and the gadolinium enhancement effect is weaker in the tumor than in normal uterine endometrium and normal uterine smooth muscle. Owing to remarkable advancements in medical technology, T2-weighted MRI can detect small tumors. However, the usefulness of T2-weighted MRI in all gynecologic tumors remains uncertain. Therefore, this case report aimed to highlight the importance of histopathologic examinations based on surgical specimens. When contrast-enhanced MRI fails to provide medical evidence of a benign tumor, appropriate surgical treatment must be the next step. A definitive diagnosis can only be made via histopathology examination of the surgical specimen.

## Figures and Tables

**Table 1:** Differential expressions of SMA, Caveolin1, Cyclin B, Cyclin E, LMP2, NT5DC2, CD133, and Ki-67 in human uterine mesenchymal tumors and uterine LANT-like tumor.

Mesenchymal Tumor Types	Age Years	n	Protein Expression *							
			SMA	CAV1	CCNB	CCNE	LMP2	NT5DC2	CD133	Ki-67
Normal	30s–80s	76	+++	−	−	−	+++	−	−	−
Leiomyoma (LMA) (Ordinally leiomyoma) (Cellular leiomyoma)	30s–80s	40 (30) (10)	+++	++	−/+	−/(+)	+++	−/+	−	+/−
			+++	++	−/+	−	+++	−/+		+/−
			++	++	−/+	−/(+)	++	−/+		+/−
STUMP	40s–60s	12	++	++	+	−/+	−/+	−/+	NA	+/+++
Bizarre Leiomyoma	40s–50s	4	++	++	−/+	+	Focal+	+	NA	+
Intravenous LMA	50s	3	++	++	+	+	−	NA	++	+
Benign metastasizing	50s	1	++	++	+	++	−	NA	NA	++
LG-ESS ^#^	40s–50s	2	+++	++	++	+++	+++	NA	NA	++
Leiomyosarcoma	30s–80s	54	−/+	+	++	+++	−/+	++	++	++/+++
Rhabdomyosarcoma	10s, 50s	2	NA	++	−/+	+++	+++	NA	NA	NA
U.LANT ^#^-like tumour	40s	1	++	+	NA	++	−	NA	NA	−

* Staining score of expression of SMA, CAV1 (Caveorin 1), CCNB (Cyclin B), CCNE (Cyclin E), LMP2 (low molecular protein 2), NT5DC2 (5’-Nucleotidase Domain Containing 2) and Ki-67 from results of IHC experiments. Protein expression *; estimated-protein expressions by immunoblot analysis, immunohistochemistry (IHC) and/or RT-PCR (quantitative-PCR), −/+; partially positive (5% to 10% of cells stained), Focal+; Focal-positive (focal or sporadic staining with less than 5% of cells stained), ++; staining with 5% or more, less than 90% of cells stained, +++; diffuse-positive (homogenecus distribution with more than 90% of cells stained), −; negative (no stained cells). U.LANT-like tumour; uterine leiomyomatoid angiomatous neuroendocrine tumour-like tumour, LMP2, cyclin E, caveolin1, NT5DC2, CD133, Ki-67. STUMP (Smooth muscle tumor of uncertain malignant potential). Cyclin E, LMP2, Caveolin1 are potential biomarker for human uterine mesenchymal tumors. LG-ESS^#^, low-grade endometrial stromal sarcoma, LANT^#^, leiomyomatoid angiomatous neuroendocrin tumour (LANT) is described as a dimorphic neurosecretory tumor with a leiomyomatous vascular component. NA; no answer.

## Data Availability

The data supporting the findings of this study are available from the corresponding author upon reasonable request.

## Supplementary Materials

The following are available online at https://www.mdpi.com/article/10.3390/cimb45080390/s1, Supplementary Material S1: Tissue Collection; Figure S1: Suspicion of subendometrial tumor and ovarian cyst; Figure S2: Cut surface of the excised the uterus corpus, fallopian tube and ovary; Table S1: Pathogenic variants of patient’s tumor and human uterine leiomyosarcomas; Table S2: In 2022, the number of patients with uterine leiomyoma or uterine sarcoma in our medical institution.

## Abbreviations

α-SMA: α-smooth muscle actin; CA19-9: carcinoma antigen 19-9; ATRX: ATRX chromatin remodeler; CA: caveolin; CNNB: cyclin B; CCNE 1: cyclin E; DAB: 3;39′-diaminobenzidine tetrahydrochloride hydrate; Ep2d: echo-planar two-dimensional sequence; ERBB2: erb-b2 receptor tyrosine kinase 2; ESS: endometrial stromal sarcoma; HHF-35: hyperinsulinemic hypoglycemia familial-35; HG: high-grade; HG-ESS: high-grade endometrial stromal sarcoma; IHC: immunohistochemistry; LMP2: large multifunctional protease 2: LG: ow-grade; LG-ESS: low-grade endometrial stromal sarcoma; LMS: leiomyosarcoma; MDM2: mouse double minute 2 Homolog; MRI: magnetic resonance imaging; mem; MED12: mediator complex subunit 12; T2WI: T2-weighted imaging; TCS2: Trancher Collins syndrome 2; TMB: tumor mutation burden; TP53: tumor protein 53; UL: uterine leiomyoma; WHO: World Health Organization.
