# Supplementary material for: Characteristic of Endometrial Stromal Sarcoma by Algorithm of Potential Biomarkers for Uterine Mesenchymal Tumor
Source: Curr Issues Mol Biol. 2023 Jul 25;45(8):6190–201. doi: 10.3390/cimb45080390 (PMC10453212; doi:10.3390/cimb45080390)
Supplement: Supplementary file 1 [file cimb-45-00390-s001.zip › cimb-2501848-supprementary material.pdf]

Supplementary Materials

Characteristic of Endometrial stromal sarcoma by algorithm of potential biomarkers for uterine mesenchymal tumor

Takuma Hayashi, Kenji Sano, Nobuo Yaegashi, Kaoru Abiko, Ikuo Konishi

Supplementary material S1

Tissue Collection. A total of 101 patients between 32 and 83 years of age and diagnosed as having smooth muscle tumors of the uterus were selected from pathological files. Serial sections were cut from at least 2 tissue blocks from each patient for hematoxylin and eosin staining and immunostaining. All tissues were used with the approval of the Ethical Committee of Shinshu University after obtaining written consent from each patient. The pathological diagnosis of uterine smooth muscle tumors was performed using established criteria (Hendrickson and Kempson, 1995) with some modification. Briefly, usual leiomyoma (usual LMA) was defined as a tumor showing typical histological features with a mitotic index (MI) [obtained by counting the total number of mitotic figures (MFs) in 10 high-power fields (HPFs)] of <5 MFs per 10 HPFs. Cellular leiomyoma (cellular LMA) was defined as a tumor with significantly increased cellularity (>2000 myoma cells / HPF) and a MI<5, but without cytologic atypia. Bizarre leiomyoma (BL) was defined as a tumor either with diffuse nuclear atypia and a MI<2 or with focal nuclear atypia and a MI<5 without coagulative tumor cell necrosis. A tumor of uncertain malignant potential (UMP) was defined as tumor with no mild atypia and a MI<10 but with coagulative tumor cell necrosis. Leiomyosarcoma (LMS) was diagnosed in the presence of a MI>10 with either diffuse cytologic atypia, coagulative tumor cell necrosis, or both. Of the 105 cases of smooth muscle tumors, 52 cases were diagnosed as LMA, three cases were BL, two cases were intravenous leiomyomatosis, 58 cases were uterine LMS, two cases of LG-ESS, one case was uterine LANT-like tumor, and two cases were uterine rhabdomyosarcoma. Of the 58 cases of LMS, 48 cases were histologically of the spindle-cell type and 10 cases were of the epithelioid type. The clinical stage of the LMS patients was stage I in 11 cases, stage II or III in 31 cases, and stage IV in 16 cases. Protein expression studies with cervix epithelium and carcinoma tissues were performed using tissue array (Uterus cancer tissues, AccuMax Array, Seoul, Korea). Details about tissue sections are indicated in manufacture’s information (AccuMax Array).

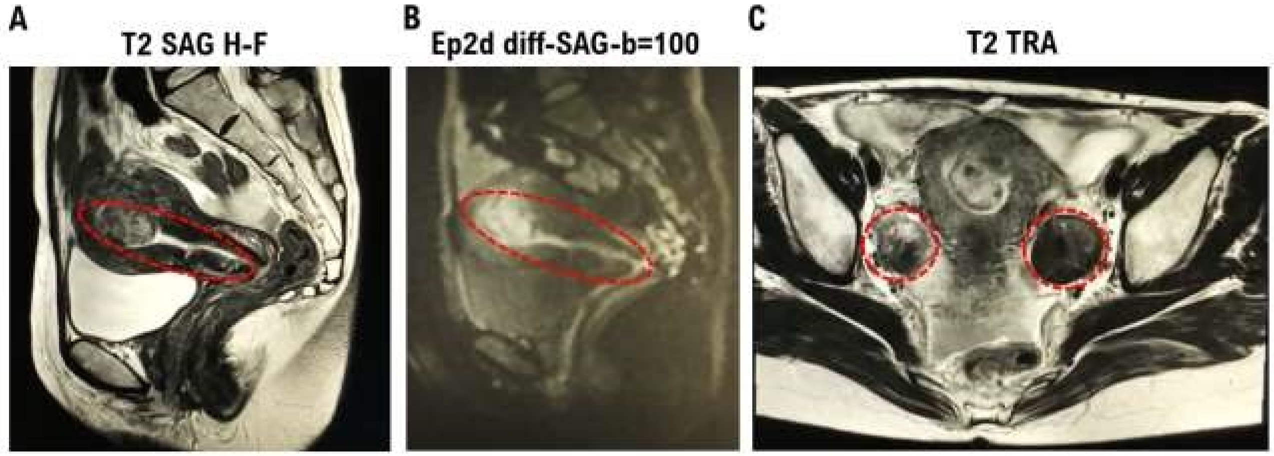

Supplementary Figure S1. Suspicion of subendometrial tumor and ovarian cyst.
A. contrast-enhanced MRI T2 SAG H-F shows a moderate to low-intense mass, suggesting submucosal uterine leiomyoma. **B.** contrast-enhanced MRI Ep2d diff-SAG-b=100 shows a mass with high signal. Subendometrial tumors are circled in red dotted lines. **C.** Contrast-enhanced MRI T2WI imaging showed bilateral ovarian shading. Contrast-enhanced MRI T2WI (TRA) imaging showed bilateral ovarian cystic lesions with fs-T2WI high signal (30 mm size signal in right ovary, 35 mm size signal in left ovary). The bilateral ovarian cystic lesions are circled in red dotted lines.

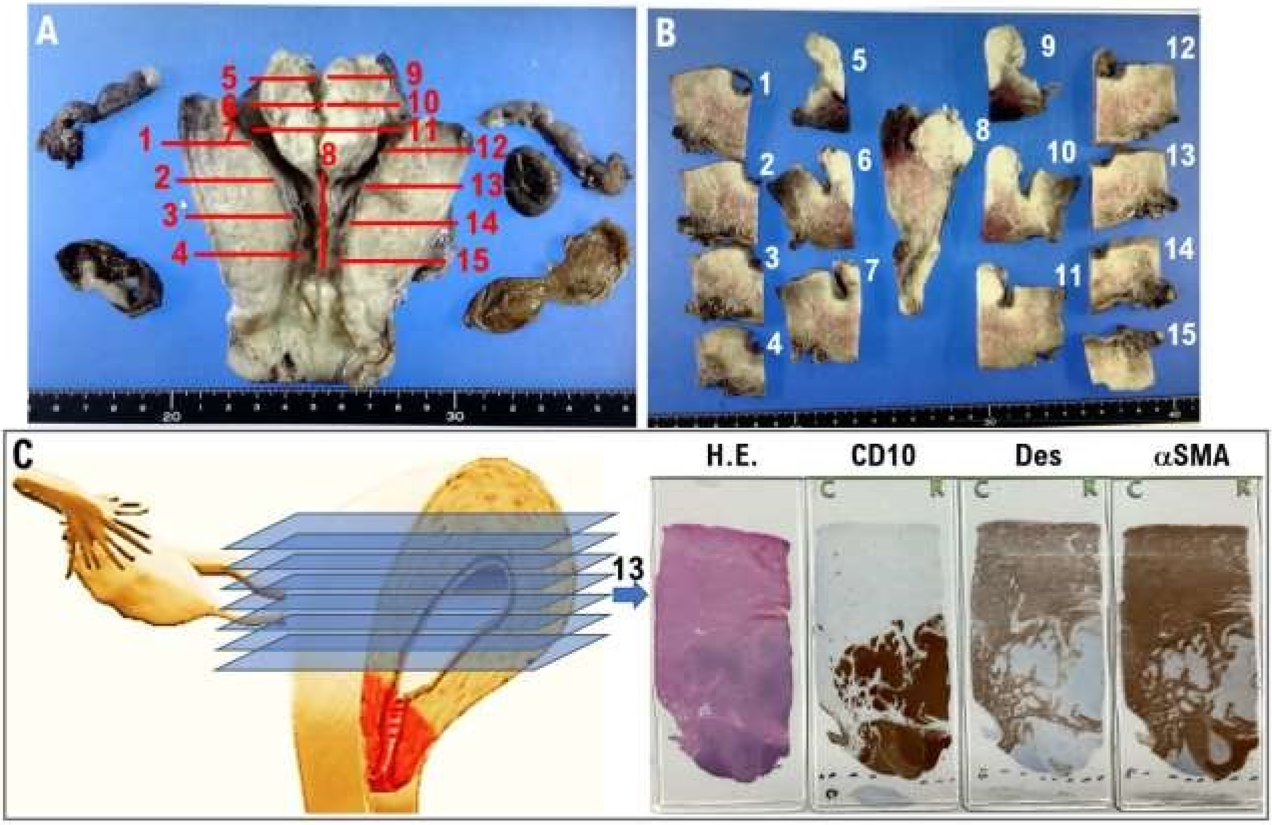

Supplementary Figure S2.
A. and B. Cut surface of the excised the uterus corpus, fallopian tube and ovary: Macroscopic findings of the excised tissue are shown below. A solid white nodule measuring 4.0 cm in size is observed within the wall of the uterine corpus. **C.** Immunohistochemical staining revealed strong expression of CD10, a molecular marker for cells that make up the endometrial stromal tissue, in the

patient's uterine tumor. However, CD10 expression was not detected in normal uterine smooth muscle tissue. As a result of immunohistochemical staining, strong expression of desmin, a molecular marker of muscle cells, was not observed in the patient's uterine tumor. However, strong expression of Desmin was observed in normal uterine smooth muscle tissue and uterine leiomyosarcoma tissue. Strong expression of α-SMA, a molecular marker for smooth muscle cells, was not found in the patient's uterine tumor. H.E.;

Result from examination of FoundationOne® CDx tissue

Genome status	Results
microsatellite status	stable
mutation burden (TMB)	5 Muts/Mb
LOH	2.1%
Pathogenic variants	*ERBB2*; copy number 41, *TCS2* Q1148* (allele frequency 43.4%), *TP53* t211I (allele frequency 54.9%)

Possible pathogenic variants of human utrine leiomyosarcoma

Genome status	Results
microsatellite status	High depending on the patient
mutation burden (TMB)	High depending on the patient
LOH	< 2.0%
Pathogenic variants	ATRX, CCNE1, LMP2/β1i, MDM2, TP53, Rb,

Supplementary Table S1. Pathogenic variants of patient's tumor and human uterine leiomyosarcomas. Pathogenic variants of patient's tumor and human uterine leiomyosarcomas are identified by the FoundationOne® CDx tissue examination (Foundation Medicine, Inc., Cambridge, MA, USA) with patient tissues resected by surgical treatment.

The number of patients with uterine leiomyoma or uterine sarcoma

Uterine Leiomyomas (uLMA)		
Surgical procedures		cases/year (2022)
Uterine Leiomyoma enucleation	laparotomy	4
Uterine Leiomyoma enucleation	laparoscopic	26
Simple hysterectomy	laparotomy	12
Simple hysterectomy	laparoscopic	50
Simple hysterectomy	robot assisted	18
Total		110

Uterine sarcoma (leiomyosarcoma, uterine endometrial stromal sarcoma)	
	cases/year (2022)
Uterine leiomyosarcoma	6
Uterine endometrial stromal sarcoma	10
Total	16

Supplementary Table S2. In 2022, the number of patients with uterine leiomyoma or uterine sarcoma in our medical institution.
